# Multinodular Goiter Spontaneous Hemorrhage in ESRD Patients Result in Acute Respiratory Failure: A Case Report

**DOI:** 10.1097/MD.0000000000002777

**Published:** 2016-02-12

**Authors:** Wen-Hui Lei, Chu-Xiao Shao, Jun Xin, Jie Li, Ming-Feng Mao, Xue-Ping Yu, Lie Jin

**Affiliations:** From the Department of Nephrology, The Fifth Affiliated Hospital of Wenzhou Medical University, Lishui, Zhejiang Province (W-HL, JL, M-FM, LJ); Department of Urology, The First Hospital of Quanzhou Affiliated to Fujian Medical University, Quanzhou, Fujian (JX); Department of General Surgery, The Fifth Affiliated Hospital of Wenzhou Medical University, Lishui, Zhejiang Province (C-XS); Department of Infection Diseases, The First Hospital of Quanzhou Affiliated to Fujian Medical University, Quanzhou, Fujian (X-PY).

## Abstract

Euthyroid multinodular goiters may lead to acute respiratory failure caused by airway obstruction or laryngotracheal compression. Here, we present a case admitted to the nephrologist with multinodular goiter spontaneous hemorrhage along with respiratory failure and end-stage renal disease (ESRD), which required urgent surgical intervention.

We report the case of a 63-year-old woman with ESRD who presented with a rapidly enlarging nodular goiter resulting in acute respiratory failure. Endotracheal intubation and emergency partial thyroidectomy were performed, revealing multinodular goiter spontaneous hemorrhage by postoperative histopathology. Several cases of benign goiters necessitating endotracheal intubation have been reported. Goiters are among the rare diagnoses in patients consulting at our institution's Nephrology.

This case illustrates that ESRD patients with benign goiter may lead to acute respiratory failure due to airway obstruction or laryngotracheal compression. It was found in agreement with previous reports. This case highlights that ESRD patient at risk of this life threatening complication such as multinodular goiter hemorrhage should be managed with elective thyroidectomy to reduce morbidity and mortality.

## INTRODUCTION

The differential diagnoses of massive, rapidly growing anterior neck masses are mainly malignant tumor such as lymphomas or thyroid carcinomas.^1,2^ This is an uncommon case of an end-stage renal disease (ESRD) patient presenting with a history and clinical findings suggestive of malignancy, but revealed a benign goiter hemorrhage into a thyroid nodule leads to a rapidly expanding haematoma on histological examination.

Multinodular goiter hemorrhage is an uncommon finding in ESRD patients. In this article, we report a case of benign goiter spontaneous hemorrhage leading to airway obstruction which was treated with urgent surgical intervention.

## CASE REPORT

In April 2015, a 63-year-old woman with ESRD hemodialysis for 6 years presented to nephrologist with a 1-day history of large neck swelling, hoarseness, and progressive dysphagia. Review of history revealed a 1-year history of nodular goiter, initially grape-sized on either lobe with no hyperthyroid or hypothyroid symptoms. The patients had a history of undergoing hemodialysis for 6 years. The primary renal diseases were IgA nephropathy. She did not take any anticoagulant or aspirin. The patient had no other remarkable medical history, and had no family history of thyroid malignancies or goiters. Arterial blood gas analysis (ABGA) was performed and respiratory acidosis was detected due to hypoxia with pH of 7.29, oxygen partial pressure (pO2) of 65, carbon dioxide partial pressure (pCO2) of 65 mmHg, oxygen saturation of blood (sO2) of 80%, and bicarbonate (HCO3) concentration of 30.1 mEq/L.

**FIGURE 1:**
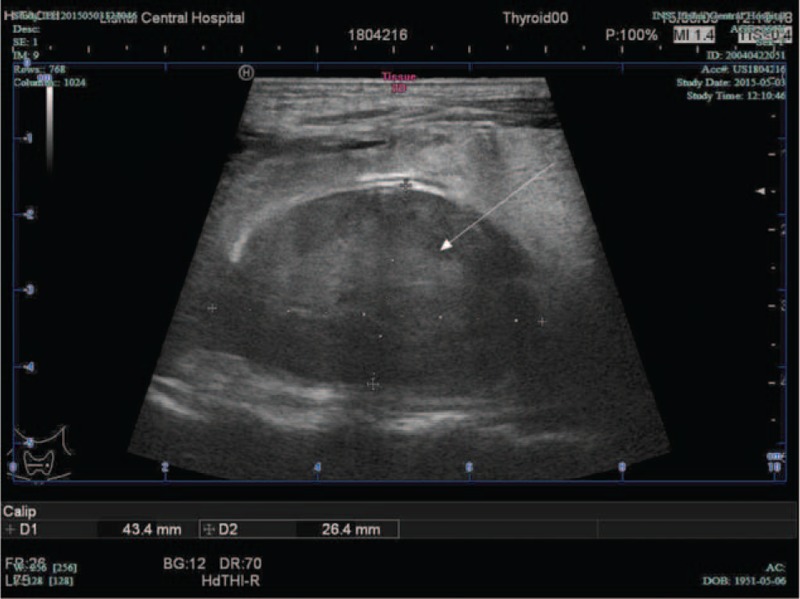
Ultrasonography illustrated a well-defined mass.

**FIGURE 2:**
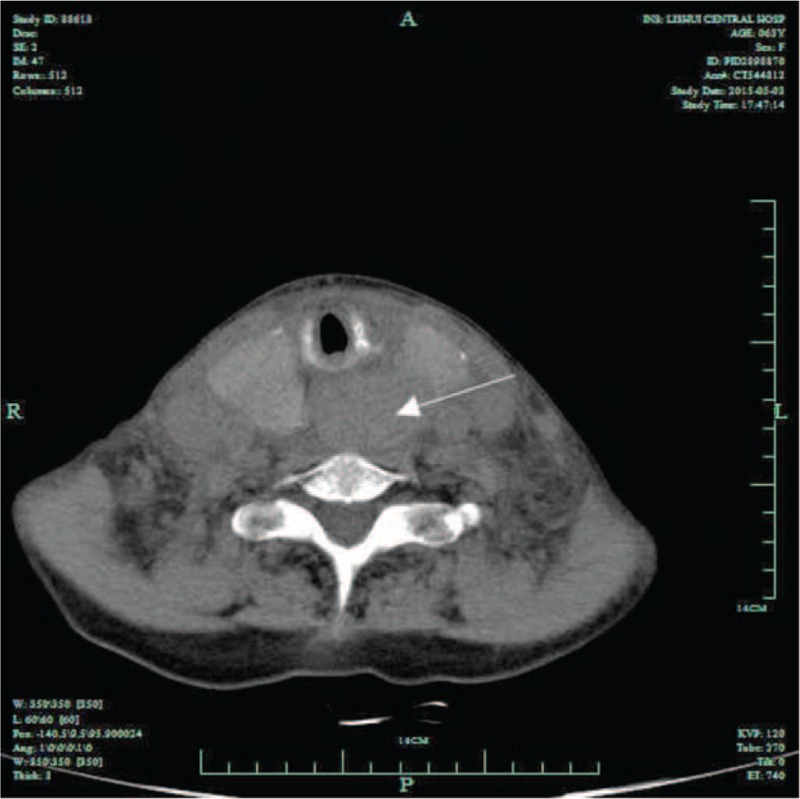
CT showed a well-defined mass compressing the trachea and esophagus.

The patient had difficulties for swallowing and stable vital signs when admitted to hospital. Pertinent physical examination findings included a markedly large nodular goiter without any cervical or axillary lymphadenopathies; respiratory sounds were normal on chest auscultation. The blood platelet and coagulation function was normal. Thyroid function tests (free T4 and thyroid stimulating hormone (TSH)) were also normal. Initial analyses of complete blood cell count, arterial blood gas, electrolyte levels were normal. Ultrasonography of the thyroid gland revealed multiple thyroid nodules and a well-defined mass behind the trachea (Figure 1). The mass was consistent with computed tomographic (CT) scan of the neck (Figure 2). The patient was given advice for surgery immediately considering of the danger of upper airways obstruction. But the patient and family refused thyroid surgery.

**FIGURE 3:**
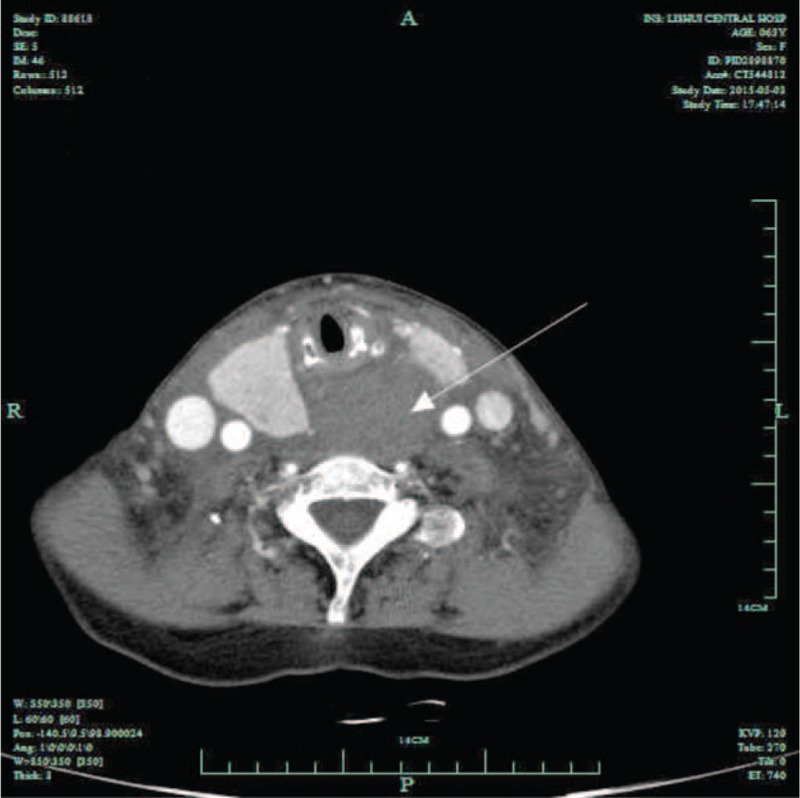
CT scans demonstrated the mass had enlarged and was nonenhancing.

**FIGURE 4:**
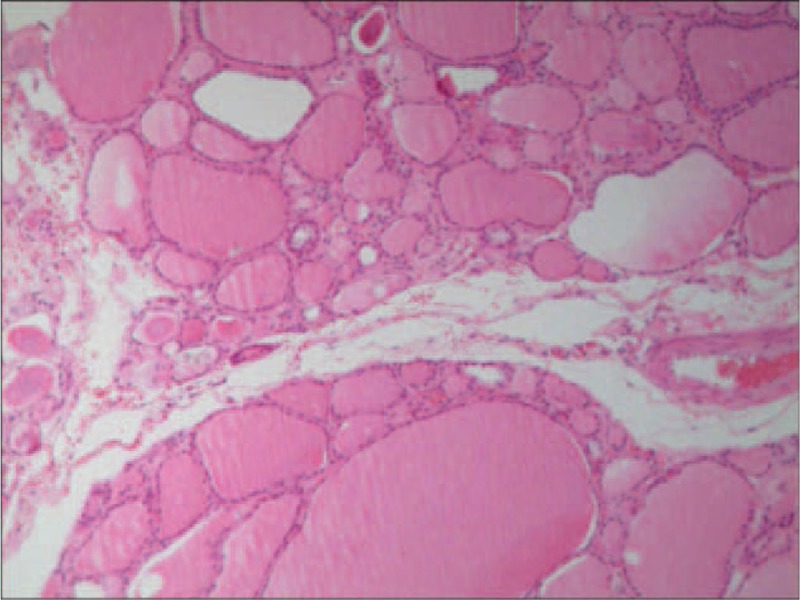
Multinodular goiter was confirmed by postoperative histopathology (EvG stain, original magnification ×100).

**FIGURE 5:**
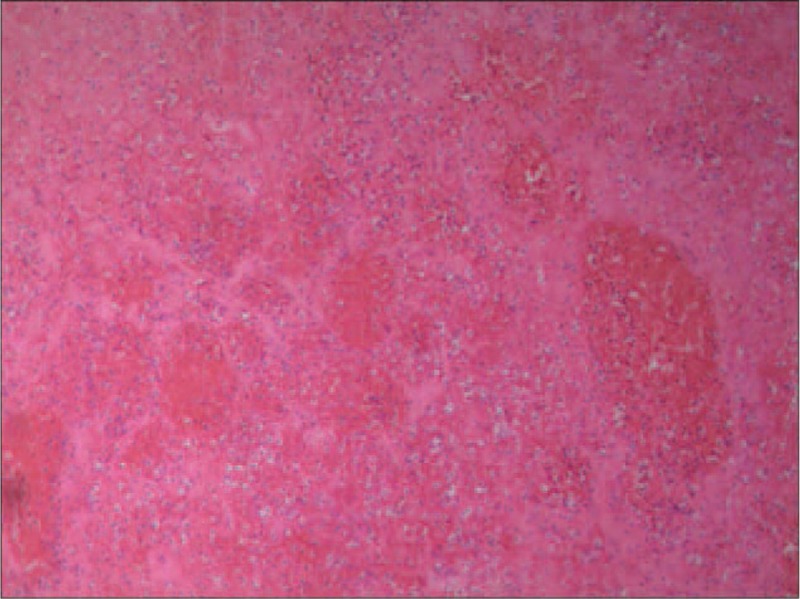
Pathological outcomes showed sludged blood and infiltration of inflammatory cells (EvG stain, original magnification ×100).

Twenty-four hours after being admitted to the hospital, the patients began to have a symptom of worsening shortness of breath and dysphagia. Arterial blood gas analysis was performed and respiratory acidosis was detected due to hypoxia with pH of 7.28, oxygen partial pressure (pO2) of 67 mm Hg, carbon dioxide partial pressure (pCO2) of 55 mm Hg, oxygen saturation of blood (SaO2) of 82%. Contrast-enhanced CT scans were used to check the mass behind tracheal. On arterial phase contrast-enhanced computed tomography image, the mass had enlarged and was nonenhancing. The CT demonstrated that the mass was compressing both her airway and esophagus (Figure 3). Endotracheal intubation was performed immediately. On intubation, the larynx was compressed and a 6.0 Fr endotracheal tube (ET) was passed. With the aforementioned history and presentation, primary consideration prior to surgery was a neck abscess or thyroid malignancy, with the patient's sex, massive and relatively rapid increase in goiter size and severe airway compromise. Despite operational risks to the end-stage renal disease patient, an urgent partial thyroidectomy was performed successfully. Multinodular goiter spontaneous hemorrhage was confirmed by postoperative histopathology (Figures 4 and 5).

Her intraoperative course was unremarkable. Postoperative CT scan showed the left thyroid lobe and the haematoma was excised. She was weaned off mechanical ventilation and extubated on the fourth postoperative day. The patient was discharged on postoperative day 10. On follow-up, the patient had complete resolution of compressive symptoms and hoarseness. The blood calcium results were in normal ranges after the partial thyroidectomy. Repeat neck ultrasonography after 1 month showed no mass in the neck. Informed consent was given by the patient for using her clinical data.

## DISCUSSION

Goiters are among the most common diagnoses in patients consulting at institution's endocrinology and otorhinolaryngology outpatient clinics. Prevalence rates vary widely among populations probably due to differences in iodine sufficiency, genetics, and even the method of diagnosis used. A review of 624 thyroidectomies shows that multinodular goiter (MNG) was common in females and MNG appears to be associated with long standing of iodine deficiency.^3^ Some literature^4^ reported that a high altitude area and environmental, urinary iodine status was associated with goiter and goiter prevalence. It has been reported^5,6^ a high incidence of goiter and nodules on thyroid ultrasonography in patients with end-stage renal disease (ESRD). Jusufovic and Hodzic^7^ also reported HD patients were with an increased thyroid volume and thyroid diseases are much more common in patients on HD compared with the general population. This may be due to the decreased clearance of the inorganic iodides, causing a hypertrophic effect on the thyroid gland tissue leading to goiter.^8^ So screening for abnormal thyroid morphology is important for HD patients.^9,10^

In such patients with goiters, it is generally believed that goiter affects the oesophagus as well as the trachea in a considerable number of people. Benign goiter with acute respiratory failure is a common situation. Several cases of goiters lead to respiratory failure had been reported before.^11–17^ However, a limited number of cases^18,19^ reported that significant hemorrhage into a thyroid cyst or nodule can lead to a rapidly expanding haematoma with subsequent airway compromise. Some literature reports that hemorrhage into a thyroid nodule does not commonly cause severe symptoms and extensive hemorrhage is relatively rare.^20,21^ The goite Hemorrhage commonly occur after exertion or trauma (such as fine needle aspiration).^19^ But in this case, multinodular goiter hemorrhage was found to be the result of spontaneous hemorrhage by postoperative histopathology.

In the spontaneous thyroid hemorrhage, however, the mechanism is unclear. Venous bleeding is the most likely explanation for hemorrhage in goiters.^22^ The literature reported^23^ that Valsalva maneuver may lead to thyroid hemorrhage as it increased venous pressure. Therefore, an associated external event is often found in spontaneous hemorrhage cases, such as seemingly insignificant straining at defecation, coughing, and crying. In the present case, dialysis patients have several platelet defects and receive heparin during their dialysis treatment that contributes to their coagulopathy may increase the risk of hemorrhage in the patient. Further studies are needed to understand more about the multinodular goiter hemorrhage in ESRD patients. What is more, the patient was first presented to the nephrologist, so it is easy for the nephrologist to neglect the potential danger of goiter hemorrhage leading to respiratory failure.

Securing the sustained airway opening of the patient has a priority in the treatment of the multinodular goiter hemorrhage which causes respiratory failure. Surgical intervention should be planned after the endotracheal intubation was completed. However, we believe that a conservative treatment could be a safe way to treat the patient if the thyroid gland hematoma is stable without airway compromise. However, when an airway obstruction appears, emergency surgery or early intubation is recommended,^24^ as in the present case.

ESRD patients with MNG hemorrhage are just rare cases in nephrology department. In this case, it highlights that ESRD patients at risk of life-threatening complications of thyroid disease, such as tracheal compression and airway obstruction, should be managed with early surgical intervention. Thus, in chronic hemodialysis (HD) patients with neck swelling and acute respiratory failure, if other common causes are not identified spontaneous hemorrhage in multinodular goiter should be kept in mind. Surgery is our preferred treatment because of the diagnostic dilemma of tumors and the potential mortality of massive hematomas in ESRD patients.

## CONCLUSION

In summary, this case study reports a successful surgical intervention on respiratory failure secondary to MNG spontaneous hemorrhage in ESRD patients. However, life-threatening complications caused by MNG hemorrhage in ESRD patients is a rare case in nephrology department. Thus, it is important for the nephrologist to take into account the potential danger of MNG spontaneous hemorrhage leading to respiratory failure in ESRD patients.

## References

[1] Lee J Seol MY Jeong S A metabolic phenotype based on mitochondrial ribosomal protein expression as a predictor of lymph node metastasis in papillary thyroid carcinoma. *Medicine* 2015; 94:e380. 25590838 10.1097/MD.0000000000000380 PMC4602546

[2] Lee J Jeong S Park JH Aberrant expression of COT is related to recurrence of papillary thyroid cancer. *Medicine* 2015; 94:e548. 25674762 10.1097/MD.0000000000000548 PMC4602754

[3] Qureshi IA Khabaz MN Baig M Histopathological findings in goiter: a review of 624 thyroidectomies. *Neuro Endocrinol Lett* 2015; 36:48–52. 25789588

[4] Omar MS El-Sayed Desouky D Environmental, urinary iodine status and prevalence of goitre among schoolchildren in a high altitude area of Saudi Arabia. *Pak J Med Sci* 2015; 31:414–419. 26101502 10.12669/pjms.312.6637 PMC4476353

[5] Da Costa AB Pellizzari C Carvalho GA High prevalence of subclinical hypothyroidism and nodular thyroid disease in patients on hemodialysis. *Hemodial Int* 2016; 20:31–37. 26246426 10.1111/hdi.12339

[6] Kaptein EM Quion-Verde H Chooljian CJ The thyroid in end-stage renal disease. *Medicine* 1988; 67:187–197. 3259281 10.1097/00005792-198805000-00005

[7] Jusufovic S Hodzic E Role of chronic hemodialysis in thyroid gland morphology disorders. *Med Arh* 2011; 65:327–329. 22299290 10.5455/medarh.2011.65.327-329

[8] Basu G Mohapatra A Interactions between thyroid disorders and kidney disease. *Indian J Endocrinol Metab* 2012; 16:204–213. 22470856 10.4103/2230-8210.93737 PMC3313737

[9] Lebkowska U Malyszko J Mysliwiec M Thyroid function and morphology in kidney transplant recipients, hemodialyzed, and peritoneally dialyzed patients. *Transplant Proc* 2003; 35:2945–2948. 14697946 10.1016/j.transproceed.2003.10.066

[10] Lin JD Hsueh C Chao TC Long-term follow-up of the therapeutic outcomes for papillary thyroid carcinoma with distant metastasis. *Medicine* 2015; 94:e1063. 26131826 10.1097/MD.0000000000001063 PMC4504566

[11] Tunc M Sazak H Karlilar B Coexistence of obstructive sleep apnea and superior vena cava syndromes due to substernal goitre in a patient with respiratory failure: a case report. *Iran Red Crescent Med J* 2015; 17:e18342. 26082848 10.5812/ircmj.17(5)2015.18342 PMC4464379

[12] Tsakiridis K Visouli AN Zarogoulidis P Resection of a giant bilateral retrovascular intrathoracic goiter causing severe upper airway obstruction, 2 years after subtotal thyroidectomy: a case report and review of the literature. * J Thorac Dis* 2012; 4 Suppl 1:41–48. 23304440 10.3978/j.issn.2072-1439.2012.s004 PMC3537381

[13] Sharma A Naraynsingh V Teelucksingh S Benign cervical multi-nodular goiter presenting with acute airway obstruction: a case report. *J Med Case Reports* 2010; 4:258. 10.1186/1752-1947-4-258 PMC2924860 20698947

[14] Sorensen JR Hegedus L Kruse-Andersen S The impact of goitre and its treatment on the trachea, airflow, oesophagus and swallowing function. A systematic review. *Best Pract Res Clin Endocrinol Metab* 2014; 28:481–494. 25047200 10.1016/j.beem.2014.03.002

[15] Bayhan Z Zeren S Ucar BI Emergency thyroidectomy: due to acute respiratory failure. *Int J Surg Case Rep* 2014; 5:1251–1253. 25437688 10.1016/j.ijscr.2014.11.012 PMC4276272

[16] Garingarao CJ Anonuevo-Cruz C Gasacao R Acute respiratory failure in a rapidly enlarging benign cervical goitre. *BMJ Case Rep* 2013; 10:e1136. 10.1136/bcr-2013-200027 PMC3736632 23878293

[17] Ioannidis O Dalampini E Chatzopoulos S Acute respiratory failure caused by neglected giant substernal nontoxic goiter. *Arq Bras Endocrinol Metabol* 2011; 55:229–232. 21655873 10.1590/s0004-27302011000300009

[18] Vijapurapu R Kaur K Crooks NH A case of airway obstruction secondary to acute haemorrhage into a benign thyroid cyst. *Case Rep Crit Care* 2014; 2014:372369. 25215246 10.1155/2014/372369 PMC4158169

[19] Hor T Lahiri SW Bilateral thyroid hematomas after fine-needle aspiration causing acute airway obstruction. *Thyroid* 2008; 18:567–569. 18397159 10.1089/thy.2007.0363

[20] Chan CC Awobem A Binns C A rare case of spontaneous thyroid cyst haemorrhage. *J Laryngol Otol* 2012; 126:960–962. 22874477 10.1017/S0022215112001430

[21] Paleri V Maroju RS Ali MS Spontaneous retro- and parapharyngeal haematoma caused by intrathyroid bleed. *J Laryngol Otol* 2002; 116:854–858. 12437846 10.1258/00222150260293727

[22] Testini M Gurrado A Lissidini G Emergency surgery for acute respiratory failure secondary to spontaneous thyroid hemorrhage. *Int Surg* 2008; 93:158–162. 18828271

[23] Johnson N The blood supply of the thyroid gland. II. The nodular gland. *Aust N Z J Surg* 1954; 23:241–252. 13159772 10.1111/j.1445-2197.1954.tb05052.x

[24] Testini M Logoluso F Lissidini G Emergency total thyroidectomy due to non traumatic disease. Experience of a surgical unit and literature review. *World J Emerg Surg* 2012; 7:9. 22494456 10.1186/1749-7922-7-9 PMC3383489

